# Epidemiology and Resistance Patterns of Bacterial and Fungal Colonization of Biliary Plastic Stents: A Prospective Cohort Study

**DOI:** 10.1371/journal.pone.0155479

**Published:** 2016-05-12

**Authors:** Christoph Lübbert, Karolin Wendt, Jürgen Feisthammel, Annette Moter, Norman Lippmann, Thilo Busch, Joachim Mössner, Albrecht Hoffmeister, Arne C. Rodloff

**Affiliations:** 1 Interdisciplinary Endoscopy Unit, Department of Gastroenterology and Rheumatology, Leipzig University Hospital, Liebigstr. 20, D-04103 Leipzig, Germany; 2 Division of Infectious Diseases and Tropical Medicine, Department of Gastroenterology and Rheumatology, Leipzig University Hospital, Liebigstr. 20, D-04103 Leipzig, Germany; 3 Interdisciplinary Center for Infectious Diseases, Leipzig University Hospital, D-04103 Leipzig, Germany; 4 Biofilm Center, German Heart Institute (Deutsches Herzzentrum Berlin, DHZB), Hindenburgdamm 30, D-12203 Berlin, Germany; 5 Institute for Medical Microbiology and Epidemiology of Infectious Diseases, Leipzig University Hospital, Liebigstr. 21, D-04103 Leipzig, Germany; 6 Department of Anaesthesiology and Intensive Care Medicine, Leipzig University Hospital, Liebigstr. 20, D-04103 Leipzig, Germany; Texas A&M University, UNITED STATES

## Abstract

**Background:**

Plastic stents used for the treatment of biliary obstruction will become occluded over time due to microbial colonization and formation of biofilms. Treatment of stent-associated cholangitis is often not effective because of inappropriate use of antimicrobial agents or antimicrobial resistance. We aimed to assess the current bacterial and fungal etiology of stent-associated biofilms, with particular emphasis on antimicrobial resistance.

**Methods:**

Patients with biliary strictures requiring endoscopic stent placement were prospectively enrolled. After the retrieval of stents, biofilms were disrupted by sonication, microorganisms were cultured, and isolates were identified by matrix-associated laser desorption/ionization time-of-flight (MALDI-TOF) mass spectrometry and/or biochemical typing. Finally, minimum inhibitory concentrations (MICs) were determined for various antimicrobial agents. Selected stents were further analyzed by fluorescence in situ hybridization (FISH).

**Results:**

Among 120 patients (62.5% males, median age 64 years) with biliary strictures (35% malignant, 65% benign), 113 double pigtail polyurethane and 100 straight polyethylene stents were analyzed after a median indwelling time of 63 days (range, 1–1274 days). The stent occlusion rate was 11.5% and 13%, respectively, being associated with a significantly increased risk of cholangitis (38.5% vs. 9.1%, P<0.001). Ninety-five different bacterial and 13 fungal species were detected; polymicrobial colonization predominated (95.8% vs. 4.2%, P<0.001). Enterococci (79.3%), *Enterobacteriaceae* (73.7%), and *Candida* spp. (55.9%) were the leading pathogens. *Candida* species were more frequent in patients previously receiving prolonged antibiotic therapy (63% vs. 46.7%, P = 0.023). Vancomycin-resistant enterococci accounted for 13.7%, extended-spectrum beta-lactamase (ESBL)-producing *Enterobacteriaceae* with co-resistance to ciprofloxacin accounted for 13.9%, and azole-resistant *Candida* spp. accounted for 32.9% of the respective isolates.

**Conclusions:**

Enterococci and *Candida* species play an important role in the microbial colonization of biliary stents. Therefore, empirical antimicrobial treatment of stent-associated cholangitis should be guided toward enterococci, *Enterobacteriaceae*, streptococci, anaerobes, and *Candida*. To determine causative pathogens, an accurate microbiological analysis of the extracted stent(s) may be helpful.

## Introduction

**Fig 1:**
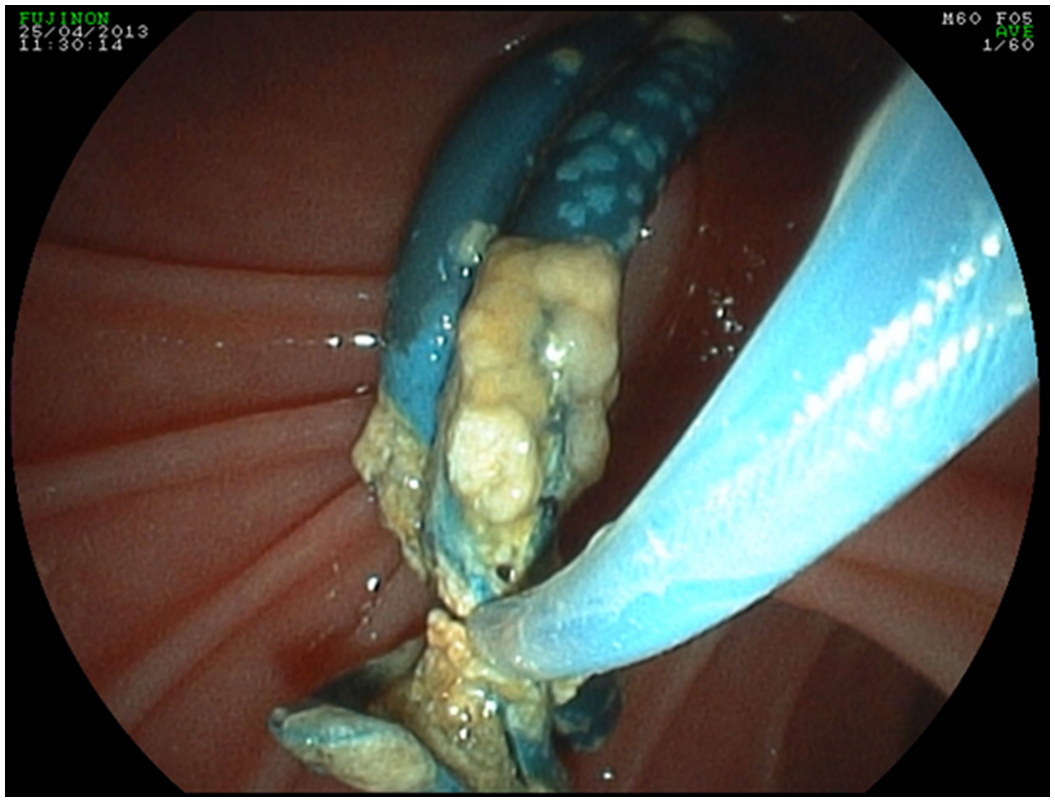
Endoscopic extraction of occluded biliary polyethylene stents (double stenting in a 52 year-old patient with biliary stenosis due to chronic pancreatitis).

Obstruction of the biliary system by malignancies, anastomotic stenosis after liver transplantation, chronic pancreatitis, or gallstones that are not immediately extractable endoscopically prevents the drainage of bile fluids from the liver and gall bladder to the small intestine and results in obstructive jaundice [1–3]. Gold standard for palliative treatment of an obstructed bile duct is the insertion of a stent by endoscopic retrograde cholangiopancreatography (ERCP) to restore bile fluid drainage [2,3]. However, sooner or later, biliary stents become colonized by microorganisms and ultimately occluded by a sludge (Fig 1) composed of bacteria, fungi, proteins, calcium bilirubinate, calcium palmitate, cholesterol, and plant fibers, leading to recurrent cholestasis or cholangitis [1,4–9]. The risk for stent occlusion depends on the indwelling time of the stent [1–3,8,10]. According to available studies, median patency of plastic stents ranges between 70 and 126 days [2,3,8,10]. Therefore, most endoscopy units perform stent exchanges at a programmed interval of three months to avoid stent occlusion [2,10,11]. Self-expanding metal stents may double the patency time as compared to straight polyethylene stents [12]. The application of antibiotics, such as ofloxacin, alone or in combination with ursodeoxycholic acid, has failed in preventing stent occlusion [13].

A broad range of studies suggest that bacterial adhesion to artificial surfaces, resulting in the formation of biofilms, drives sludge development ahead [10,14–22]. A biofilm is a structured consortium of microorganisms embedded in a self-produced polymer matrix consisting of polysaccharides, proteins, and deoxyribonucleic acid (DNA). This consortium can consist of one or more species living in a sociomicrobiological way [21]. Bacterial biofilms predispose for chronic infections because indwelling microorganisms show increased tolerance to antibiotics and disinfectant chemicals as well as resist phagocytosis and other components of the body’s defense system [21]. In a pioneering study published in 1988, Leung et al. perfused biliary stents with either bacterially contaminated or sterile bile fluid [14]. Scanning electron microscopy revealed a dense layer of bacteria and amorphous material on the surface of the stents which were perfused with bacterially contaminated bile. In contrast, this phenomenon was not observed in experiments using sterile bile only [14].

The clinical management of biofilm-associated infections is challenging because the microbes in the stent are not generally amenable to eradication employing antimicrobial chemotherapy [1]. Thus, the occluded device must be replaced endoscopically, which requires an elaborate and, from the perspective of the patient, stressful procedure. Although stent replacement may be repeated several times, stent lifetime tends to decrease progressively, presumably because relevant microbes that have adapted to the stent niche accumulate within the bile duct [1]. Biofilms developing on biliary stents usually consist of a broad and diverse spectrum of mixed microbial communities [1,17,19]. However, many bacteria of the gut microbiota may not presently be cultivated by standard laboratory procedures. Thus, existing data on biliary stent microbial communities may be biased and incomplete [1]. Notably, even available information on culturable bacteria is not static and shows a recent trend toward pathogens such as extended-spectrum beta-lactamase (ESBL)-producing *Enterobacteriaceae*, enterococci, and *Candida* species [10,23–28]. Thus, standard antimicrobial therapy concepts for patients with cholangitis, which mainly target sensitive *Enterobacteriaceae* and anaerobes, need to be reevaluated [25–28].

We performed this prospective cohort study to determine the current bacterial and fungal etiology of biofilm formation in biliary stents, with particular emphasis on antibiotic therapy and thereby exerted selective pressure. A further objective was to assess the dynamics of pathogens and the epidemiology of antimicrobial resistance compared to what was found in previous studies [10,14,17,19,20,23,24].

## Patients and Methods

### Study population and study design

From June 2013 to March 2015, 120 patients with biliary strictures and elective or emergency stent exchanges were consecutively enrolled. Stent exchanges were performed at the interdisciplinary endoscopy unit of the Leipzig University Hospital (Leipzig, Germany) by experienced endoscopic examiners (personal record of >250 ERCPs).

### Ethics approval

The study was performed in accordance with the ethical guidelines of the 1964 Declaration of Helsinki and was approved by the local ethics committee (University of Leipzig, register no. 059-13-11032013). As the study did not modify patient management, and as the data were processed anonymously, the need for informed consent was waived according to the ethics committee approval.

### Interventional endoscopic procedure

ERC was performed using standard videoduodenoscopes of the Fujinon ED-530 series (Fujinon, Japan). First, the position of the indwelling biliary stent(s) was documented by direct endoscopic visualization and x-ray. Afterwards, (a) stent(s) was (were) grasped by a snare or forceps and then extracted by complete retraction of the endoscope through the stomach, the esophagus, the pharynx, and the oral cavity. Subsequently, a 5F catheter was inserted into the biliary tract, and contrast fluid was injected. Depending on the results of previous and current cholangiograms, either (a) new stent(s) was (were) inserted, or the stent therapy was ended. The caliber of subsequent stent placements varied between 8F and 11.5F, as in the previous intervention.

### Stent characteristics and stent preparation

All extracted stents were made of either polyethylene (straight stents from Cook Medical, Ireland, or from Boston Scientific, USA) or polyurethane (double pigtail stents from Optimed, Germany). To minimize the risk of contamination, and to provide the best possible pre-analytic conditions, 3–4 centimeters of the distal end of the extracted stent(s), previously located in the intestinal lumen, were removed using sterile scissors, and immediately thereafter, stents were immersed in a sterile nutrient solution (Brain Heart Infusion Broth; Merck, Germany). Transport to the microbiology laboratory was performed on the same day, where the stent(s) was (were) worked up according to a standardized protocol. In patients with multi-stenting, only one stent was submitted for microbiological analysis.

### Definition of stent occlusion

Before starting the sonication process, an assessment of the drainage function of the stent was carried out in the microbiology laboratory. Hereby, encrusted sludge completely narrowing the stent lumen was defined as inner stent occlusion, as described previously [10].

### Sonication process

In the microbiology laboratory, the prepared stent was put into a sterile container (Bandelin, Germany) and completely covered with 50 ml of sterile Ringer’s solution. To disrupt the biofilm on the inner surface of the stent, the specimen was vortexed for 30 seconds and subsequently exposed to low-frequency (40 kHz) ultrasound for 15 minutes, as described previously [10,29,30]. Sonication was performed in an ultrasound bath specially designed for microbiological analysis (BactoSonic^®^; Bandelin, Germany). Thereafter, the container was vortexed again for 30 seconds.

### Microbiological analysis

Aliquots of the sonication fluid were cultivated on conventional solid media (sheep blood agar, blood agar, chocolate agar, Esculin agar, Endo agar, Columbia agar with and without gentamicin supplementation, CHROMagar^™^ ESBL, and Sabouraud agar) and incubated in aerobic and anaerobic atmospheres at 37°C (Heraeus Incubator Series 6000; Thermo Scientific, Germany), as described previously [10]. Aerobic cultures were incubated for at least 48 hours, and anaerobic cultures for 96 hours. All bacterial isolates were identified using a matrix-associated laser desorption/ionization time-of-flight mass spectrometer (MALDI-TOF MS; bioMérieux, France). Fungal isolates were submitted to a commercially available assimilation test (ID 32 C yeast identification system; bioMérieux, France) for species identification.

### Antimicrobial susceptibility testing

Depending on the Gram stain nature of the bacterial isolates, minimum inhibitory concentrations (MICs) were established according to International Organization for Standardization (ISO) 20776–1 for the following antimicrobial agents: penicillin G, ampicillin, oxacillin, piperacillin, ampicillin/sulbactam, piperacillin/tazobactam, cefuroxime, cefotaxime, ceftazidime, imipenem, meropenem, gentamicin, ciprofloxacin, levofloxacin, moxifloxacin, erythromycin, clindamycin, vancomycin, teicoplanin, rifampicin, linezolid, daptomycin, doxycycline, trimethoprim/sulfamethoxazole, fosfomycin, colistin, and metronidazole. Tigecycline was tested using gradient strips (Etest^®^; bioMérieux, France). E-tests were also used for fungal isolates determining MICs for amphotericin B, flucytosine, fluconazole, itraconazole, posaconazole, voriconazole, and caspofungin. Susceptibilities were assessed using breakpoints established by the European Committee on Antimicrobial Susceptibility Testing (EUCAST, www.eucast.org, 2015 edition) [31]. Phenotypic ESBL production was confirmed using appropriate E-tests (bioMérieux, France), as described previously [32].

### Visualization of biofilm formation

**Fig 2:**
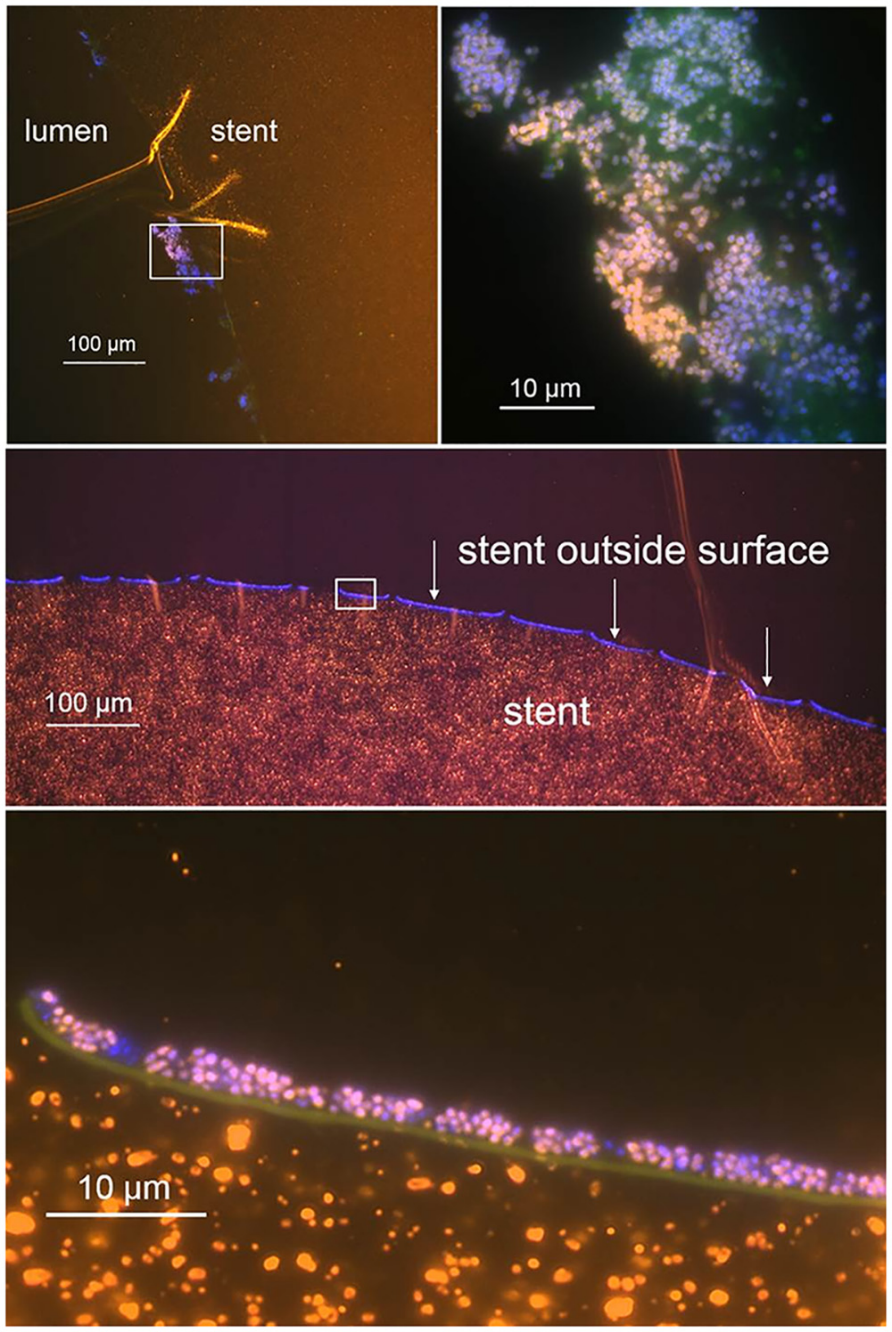
Presence of biofilm formation on a biliary polyethylene stent in a 62-year-old liver transplant recipient with anastomotic bile duct stenosis, visualized by fluorescence in situ hybridization (FISH). Using cultures, *Enteroccus faecalis* and *Escherichia coli* were detected.

In selected cases, biofilms were visualized by fluorescence in situ hybridization (FISH) (Fig 2) in the Biofilm Center of the German Heart Institute (Berlin, Germany), as described previously [33,34].

### Blood cultures

Blood cultures were collected only in patients presenting with acute cholangitis or general signs of infection.

### Statistical analysis

Statistical analysis was performed using SPSS for Windows (SPSS 20.0, IBM Corporation, Armonk, New York, USA). Numerical variables were summarized as medians, and categorical variables were given as frequencies or proportions. Confidence intervals (CI) for frequencies were calculated based on binomial distribution. Categorical data were analyzed by the chi-square test or Fisher’s exact test. For the comparison of two independent groups, the nonparametric Mann-Whitney U test was used. Independent predictors for the occurrence of enterococci or *Candida* species were identified using multiple logistic regression. For comparing the time-dependent persistence of pathogens, the log-rank test was applied. P values (two-sided) of <0.05 were considered statistically significant.

## Results

### Patients and stent characteristics

One-hundred and twenty patients (62.5% males, 37.5% females, median age 64 years) with biliary strictures (35% with malignant strictures, 20.8% after liver transplantation, 18.3% due to gallstones, 9.2% due to chronic pancreatitis, 16.7% due to other reasons) requiring endoscopic stent placement previously were prospectively enrolled in the study (Table 1). Preventive stent replacements accounted for 60.6% (129/213) of the procedures. Forty of 120 patients (33.3%) had repeating stenting procedures.

**Table 1:** Baseline characteristics of the patients and stents.

**Number of patients**	**120**		
Median age in years (range)	64 (7–91)		
Male sex (%)	75 (62.5%)		
**Reason for stent therapy**			
**Malignant disease (%)**	**42 (35%)**		
Cholangiocarcinoma (%)	26 (21.7%)		
Pancreatic cancer (%)	6 (5%)		
Other malignancy (%)	10 (8.3%)		
**Benign disease (%)**	**78 (65%)**		
Anastomotic stenosis after liver transplantation (LTx) (%)	25 (20.8%)		
Choledocholithiasis (%)	22 (18.3%)		
Chronic pancreatitis (%)	11 (9.2%)		
Idiopathic biliary stricture (%)	11 (9.2%)		
Biliary leakage (%)	5 (4.2%)		
Other reasons (%)	4 (3.3%)		
**Antibiotic therapy**			
Ceftriaxone	38 (31.7%)		
Ciprofloxacin	34 (28.3%)		
Metronidazole	31 (25.8%)		
Piperacillin/tazobactam	31 (25.8%)		
Other agents	69 (65.8%)		
**Clinical outcomes during the study period**			
Death due to biliary infection (%)	5 (4.2%)		
Death due to underlying disease (%)	24 (20%)		
Death due to other reasons (%)	9 (7.5%)		
**Total number of biliary stents**	**213**		
**Type of stent (material)**	**Double pigtail stents (polyurethane)**	**Straight stents (polyethylene)**	**P value**
Number	113	100	n/a
Indwelling time in days (range)	64 (3–1202)	58 (1–1274)	0.559
Number of treatment episodes with multiple stenting	47	35	0.324
Stent occlusion rate (%)	13/113 (11.5%)	13/100 (13%)	0.739
Signs of cholangitis (fever, abdominal pain, jaundice, pus coming out of the papilla) (%)	14/113 (12.4%)	13/100 (13%)	0.894
**Median laboratory parameters at baseline (normal range)**			
Bilirubin (μmol/L; <17.1)	13.1	12.25	0.952
ALT (μkat/L; 0.17–0.85)	0.64	0.59	0.859
AST (μkat/L; 0.17–0.85)	0.74	0.58	0.160
AP (μkat/L; 0.58–2.15)	3.61	2.44	0.007
GGT (μkat/L; 0.17–1.19)	4.71	3.79	0.071
C-reactive protein (CRP) (mg/L; <5)	17.45	18.35	0.530
WBC (GPT/L; 3.5–9.8)	6.6	7.5	0.100

Abbreviations: ALT = alanine transaminase; AP = alkaline phosphatase; AST = aspartate transaminase; GGT = gamma-glutamyl transferase; WBC = white blood cells; n/a = not applicable

**Fig 3:**
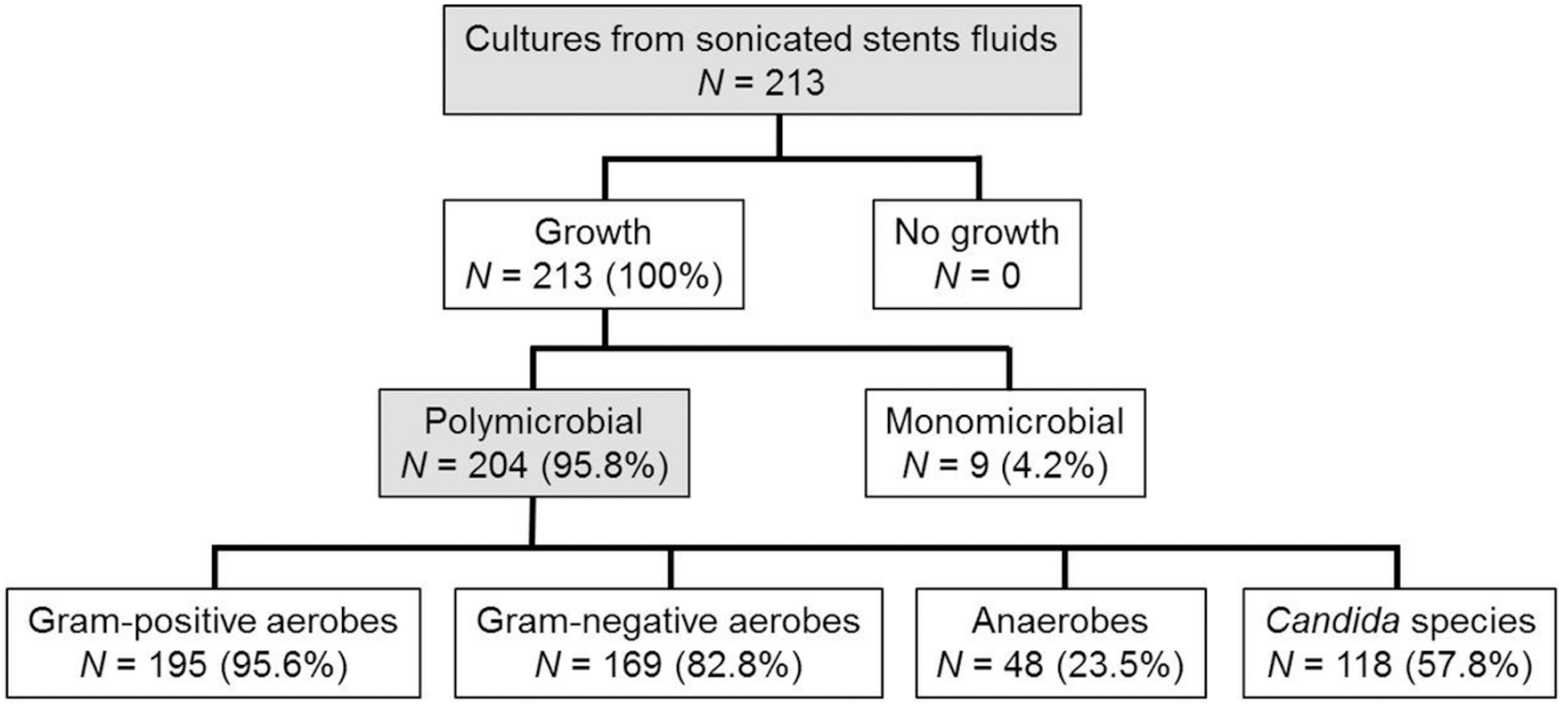
Overview of microbiological results of cultures from sonicated stent fluids. Polymicrobial colonization of biliary stents (95.8%) was significantly more common than single microbial colonization (4.2%, P <0.001) was.

In these patients, 213 biliary stents (113 double pigtail stents, 100 straight plastic stents) were microbiologically analyzed after endoscopic retrieval (Fig 3). The median time of biliary stent placement was 63 days (range, 1–1274 days). The stent occlusion rate was 11.5% (13/113; 95% CI: 6.3% to 18.9%) in double pigtail stents, and it was 13% (13/100; 95% CI: 7.1% to 21.2%) in straight polyethylene stents. The analysis of the laboratory markers at baseline revealed a statistically significant difference only for alkaline phosphatase (AP) values (*P* = 0.007), presumably due to a slightly better drainage function of straight stents compared to double pigtail stents.

The clinical cholangitis rate (defined by the occurrence of fever, abdominal pain, jaundice, pus coming out of the papilla) was 20% (24/120; 95% CI: 13.3% to 28.3%). Three patients had multiple cholangitis episodes due to repeating stenting procedures. The risk of cholangitis was significantly higher in patients with stent occlusion (38.5% vs. 9.1%, *P* <0.001). Eighty-six of the 120 patients (71.7%) received antibiotic treatments during stent insertion, mainly ceftriaxone (31.7%), ciprofloxacin (28.3%), metronidazole (25.8%), or piperacillin/tazobactam (25.8%), including combinations and sequential treatments. In patients with positive blood cultures for *Candida* species, antifungal therapy with either fluconazole, voriconazole, anidulafungin or liposomal amphotericin B was administered according to the results of the microbiological susceptibility testing.

### Detection of bacteria and fungi

All endoscopically retrieved stents showed microbial colonization, even after an indwelling time of just one day. A total of 95 different bacterial and 13 different fungal species could be detected (Table 2).

**Table 2:** Spectrum of microorganisms* isolated from biliary stents.

Bacteria			
**Gram-positive species**	**Double pigtail stents (polyurethane)**	**Straight stents (polyethylene)**	**P value**
*Enterococcus faecalis*	56/113 (49.6%)	51/100 (51%)	0.834
*Enterococcus faecium*	37/113 (32.7%)	26/100 (26%)	0.282
*Enterococcus casseliflavus*	12/113 (10.6%)	5/100 (5%)	0.204
*Enterococcus avium*	10/113 (8.8%)	5/100 (5%)	0.224
*Enterococcus gallinarum*	8/113 (7.1%)	3/100 (3%)	0.224
Enterococci (other species)	2/113 (1.8%)	4/100 (4%)	0.423
*Streptococcus anginosus*	20/113 (17.7%)	16/100 (16%)	0.741
*Streptococcus parasanguinis*	5/113 (4.4%)	6/100 (6%)	0.604
*Streptococcus oralis/mitis*	3/113 (2.7%)	7/100 (7%)	0.195
*Streptococcus constellatus*	4/113 (3.5%)	1/100 (1%)	0.374
Streptococci (other species)	8/113 (7.1%)	7/100 (7%)	0.982
*Staphylococcus aureus*	2/113 (1.8%)	3/100 (3%)	0.667
*Staphylococcus epidermidis*	5/113 (4.4%)	5/100 (5%)	1.000
*Staphylococcus haemolyticus*	2/113 (1.8%)	4/100 (4%)	0.423
Staphylococci (other species)	0/113 (0%)	3/100 (3%)	0.102
Others	10/113 (8.8%)	8/100 (8%)	0.824
**Gram-negative species**	**Double pigtail stents (polyurethane)**	**Straight stents (polyethylene)**	**P value**
*Escherichia coli*	55/113 (48.7%)	34/100 (34%)	0.030
*Enterobacter cloacae*	16/113 (14.2%)	15/100 (15%)	0.862
*Klebsiella oxytoca*	14/113 (12.4%)	10/100 (10%)	0.582
*Klebsiella pneumoniae*	9/113 (8%)	11/100 (11%)	0.448
*Citrobacter spp*.	8/113 (7.1%)	6/100 (6%)	0.751
*Hafnia alvei*	7/113 (6.2%)	4/100 (4%)	0.470
*Pseudomonas aeruginosa*	6/113 (5.3%)	6/100 (6%)	0.827
*Proteus vulgaris*	5/113 (4.4%)	6/100 (6%)	0.604
*Proteus mirabilis*	5/113 (4.4%)	2/100 (2%)	0.452
*Morganella morganii*	2/113 (1.8%)	4/100 (4%)	0.423
Others	13/113 (11.5%)	16/100 (16%)	0.340
**Anaerobes**	**Double pigtail stents (polyurethane)**	**Straight stents (polyethylene)**	**P value**
*Bacteroides spp*.	11/113 (9.7%)	6/100 (6%)	0.316
*Prevotella spp*.	6/113 (5.3%)	7/100 (7%)	0.607
*Veilonella spp*.	4/113 (3.5%)	1/100 (1%)	0.374
Others	8/113 (7.1%)	7/100 (7%)	0.982
**Fungi**	**Double pigtail stents (polyurethane)**	**Straight stents (polyethylene)**	**P value**
*Candida albicans*	55/113 (48.7%)	48/100 (48%)	0.922
*Candida glabrata*	13/113 (11.5%)	10/100 (10%)	0.724
*Candida kefyr*	5/113 (4.4%)	0/100 (0%)	0.062
*Candida tropicalis*	1/113 (0.9%)	4/100 (4%)	0.189
*Candida* (other species)	6/113 (5.3%)	4/100 (4%)	0.753
Others	3/113 (2.7%)	1/100 (1%)	0.624
**Multi-drug resistant species**	**Double pigtail stents (polyurethane)**	**Straight stents (polyethylene)**	**P value**
Vancomycin-resistant enterococci (VRE)	18/125 (14.4%)	12/94 (12.8%)	0.728
Methicillin-resistant Staphylococcus aureus (MRSA)	2/2 (100%)	1/3 (33.3%)	0.400
ESBL-producing *Enterobacteriaceae*, resistant to 3 of 4 major antibiotic classes^§^ (3MRGN)	17/129 (13.2%)	15/101 (14.9%)	0.716
Carbapenemase producing *Enterobacteriaceae*, resistant to 4 of 4 major antibiotic classes^§^ (4MRGN)	1/129 (0.8%)	0/101 (0%)	1.000
Azole-resistant *Candida* species	25/80 (31.3%)	23/66 (34.8%)	0.645

Abbreviations: MRGN = multi-drug resistant Gram-negatives

^§^cephalosporins, acylaminopenicillins, fluoroquinolones, and carbapenems

*The complete list of species included (number of isolates in brackets, in descending order of frequency):

*Enterococcus faecalis (107), Candida albicans (103), Escherichia coli (89), Enterococcus faecium (63), Streptococcus anginosus (36), Enterobacter cloacae (31), Klebsiella oxytoca (24), Candida glabrata (23), Enterococcus casseliflavus (17), Enterococcus avium (15), Pseudomonas aeruginosa (12), Enterococcus gallinarum (11), Hafnia alvei (11), Proteus vulgaris (11), Streptococcus parasanguinis (11), Staphylococcus epidermidis (10), Streptococcus oralis/mitis (10), Prevotella melaninogenica (9), Citrobacter braakii (6), Morganella morganii (6), Staphylococcus haemolyticus (6), Bacteroides vulgatus (5), Bacteroides fragilis (5), Candida kefyr (5), Candida tropicalis (5), Staphylococcus aureus (5), Streptococcus constellatus (5), Veilonella parvula (5), Citrobacter freundii (4), Lactobacillus rhamnosus (4), Neisseria subflava (4), Raoultella planticola (4), Streptococcus gallolyticus ssp. gallolyticus (4), Streptococcus salivarus spp. salivarus (4), Actinomyces odontolyticus (3), Bifidobacterium spp. (3), Enterococcus durans (3), Enterococcus hirae (3), Haemophilus parainfluenzae (3), Serratia marcescens (3), Candida colliculosa (2), Candida krusei (2), Candida parapsilosis (2), Citrobacter koseri (2), Citrobacter youngae (2), Cronobacter sakazakii (2), Enterobacter aerogenes (2), Lactobacillus plantarum (2), Prevotella buccae (2), Rothia mucilaginosa (2), Streptococcus sanguinis (2), Acinetobacter baumannii (1), Abiotrophia defectiva (1), Actinomyces viscosus (1), Aeromonoas hydrophila (1), Bacillus pumilus (1), Bacteroides uniformis (1), Bacteroides ovatus (1), Bacteroides stercoris (1), Bacteroides thetaiotaomicron (1), Candida dubliniensis (1), Candida guilliermondii (1), Candida lambica (1), Candida norvegensis (1), Clostridium perfringens (1), Corynebacterium aurimucosum (1), Corynebacterium bovis (1), Corynebacterium pseudodiphthericum (1), Corynebacterium striatum (1), Cryptococcus huminocola (1), Cryptococcus laurentii (1), Enterobacter amnigenus (1), Escherichia hermanii (1), Gemella sanguis (1), Lactobacillus casei (1), Lactobacillus acidophilus (1), Lactococcus garvieae (1), Leifsonia aquatica (1), Microbacter flavescens (1), Moraxella osloensis (1), Mycobacterium avium (1), Parabacteroides distasonis (1), Pediococcus pentosaceus (1), Prevotella denticola (1), Prevotella oris (1), Proprionibacterium avidum (1), Propionibacterium acnes (1), Providencia rettgeri (1), Pseudomonas stutzeri (1), Ralstonia insidiosa (1), Raoultella ornithinolytica (1), Rhizobium radiobacter (1), Saccharomyces cerevisiae (1), Salmonella spp. (1), Staphylococcus capitis (1), Staphylococcus saprophyticus (1), Staphylococcus vitulinus (1), Streptococcus suis (1), Streptococcus equinus ssp. zooepidemicus (1), Streptococcus gallolyticus ssp. pasteurianus (1), Streptococcus infantarius spp. coli (1), Streptococcus pneumoniae (1), Trueperella bernardiae (1), Vibrio alginolyticus (1), Yersinia pseudotuberculosis (1)*.

**Fig 4:**
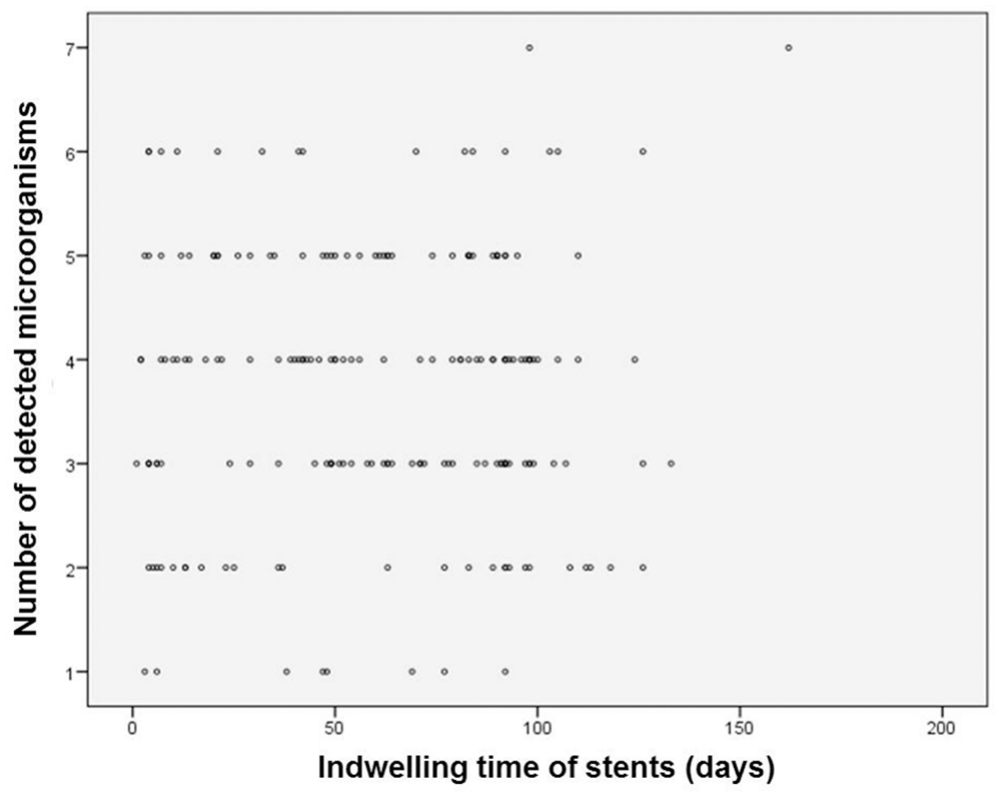
Scatter plot of stent duration in place (in days) vs. number of detected microorganisms.

Up to seven different species were detected on a single stent (Fig 4). Polymicrobial colonization (95.8%; 95% CI: 92.1% to 98%) was significantly more common than monomicrobial colonization (4.2%; 95% CI: 2% to 7.9%) was (*P* <0.001). Enterococci were detected in 79.3% (169/213; 95% CI: 73.3% to 84.6%) of all stents, *Enterobacteriaceae* in 73.7% (157/213; 95% CI: 67.3% to 79.5%), streptococci in 31.5% (67/213; 95% CI: 25.3% to 38.2%), anaerobes in 23.5% (50/213; 95% CI: 18% to 29.7%), and staphylococci in 11.3% (24/213; 95% CI: 7.4% to 16.3%). In 55.9% (119/213; 95% CI: 48.9% to 62.6%) of all cases, *Candida* species were involved. Species identification revealed *Enterococcus faecalis* as the most common organism (50.2% of all stents), followed by *Candida albicans* (48.4%), *Escherichia coli* (41.7%), *Enterococcus faecium* (29.6%), *Streptococcus anginosus* (16.9%), *Enterobacter cloacae* (14.6%), *Klebsiella oxytoca* (11.3%), and *Candida glabrata* (10.8%).

In patients with repeating stenting procedures, a clear tendency toward the establishment of the same microbial flora on consecutive stents could be demonstrated, with a concordance rate of 84% for *Enterococcus faecalis*, 83% for *Candida albicans*, 72% for *Escherichia coli*, and 60% for *Enteroccocus faecium* as the most common pathogens.

The microbiological species analysis revealed no significant difference between the microbial colonization of either polyethylene or polyurethane stents, regardless of their diameters, except for *Escherichia coli* (*P* = 0.030). Further statistically significant differences were found for colonization with *Candida* species (67.1% vs. 49.6%, *P* = 0.015), viridans group streptococci (40.8% vs. 26.3%, *P* = 0.032), and *Pseudomonas aeruginosa* (1.3% vs. 8.8%, *P* = 0.035), respectively, comparing malignant and benign biliary strictures (S1 Table).

### Matching rate of blood cultures and stent samples

In 42 of the 120 patients, a total of 58 blood cultures were obtained, of which 30 were positive. In 17 of these (56.7%; 95% CI: 37.4% to 74.5%), there was agreement with the microbiological results from biliary stents regarding the detected pathogen (the microbial species involved were *Enterococcus faecalis*, *Enterococcus faecium*, *Enterococcus avium*, *Streptococcus anginosus*, *Escherichia coli*, *Enterobacter cloacae*, *Klebsiella pneumoniae*, and *Candida albicans*), and in 11 there was complete agreement with the respective phenotypic resistance profiles.

Five of 120 patients (4.2%) died due to biliary infections with sepsis. Four of these patients were liver transplant recipients, and one patient suffered from metastatic gallbladder carcinoma. Blood cultures detected *Enterobacteriaceae (Escherichia coli*, *Klebsiella pneumoniae*, *Klebsiella oxytoca*, *Serratia marcescens)*, Enterococci *(Enterococcus faecium)* and *Pseudomonas aeruginosa* as triggering pathogens. Antimicrobial treatment was performed by carbapenems and linezolid according to the results of the microbiological susceptibility testing.

### Time dependency of stent colonization

**Fig 5:**
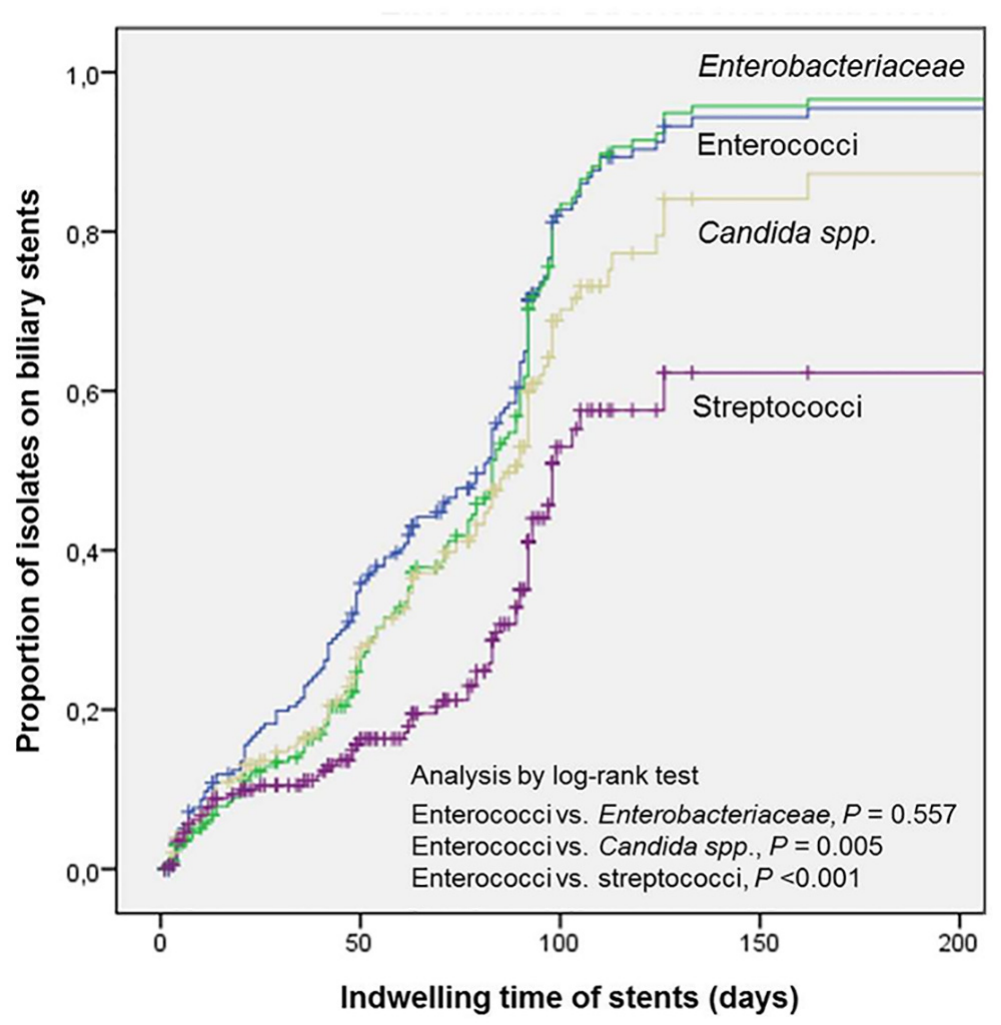
Stent duration in place (in days) versus microbiological isolates from sonicated stent fluids, grouped by predominant enterococci, *Enterobacteriaceae*, *Candida* spp., and streptococci.

The time-dependent graphical representation of stent colonizers grouped by enterococci, *Enterobacteriaceae*, *Candida* species, and streptococci showed a significantly different distribution of enterococci versus *Candida spp*. (*P* <0.001), and enterococci versus streptococci (*P* = 0.005), respectively (log-rank test) (Fig 5).

### Stent colonization with respect to antibiotic therapy

**Fig 6:**
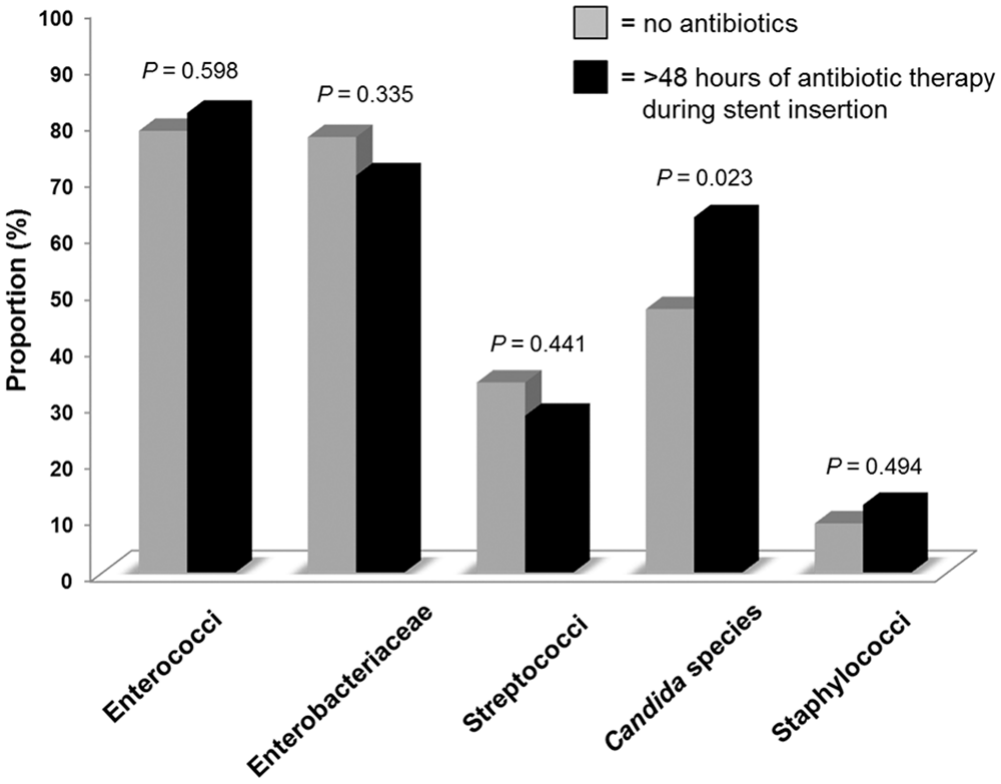
Distribution of bacterial and fungal species depending on the use of antibiotics. Stent colonization differed significantly with respect to the proportion of *Candida* species (46.7% vs. 63%, P = 0.023) in patients receiving prolonged antibiotic therapy.

Stent colonization differed significantly with respect to the proportion of *Candida* species (63% vs. 46.7%, *P* = 0.023) in patients receiving prolonged antibiotic therapy (Fig 6). In the other groups, no significant differences were found.

### Antimicrobial resistance profiles

**Fig 7:**
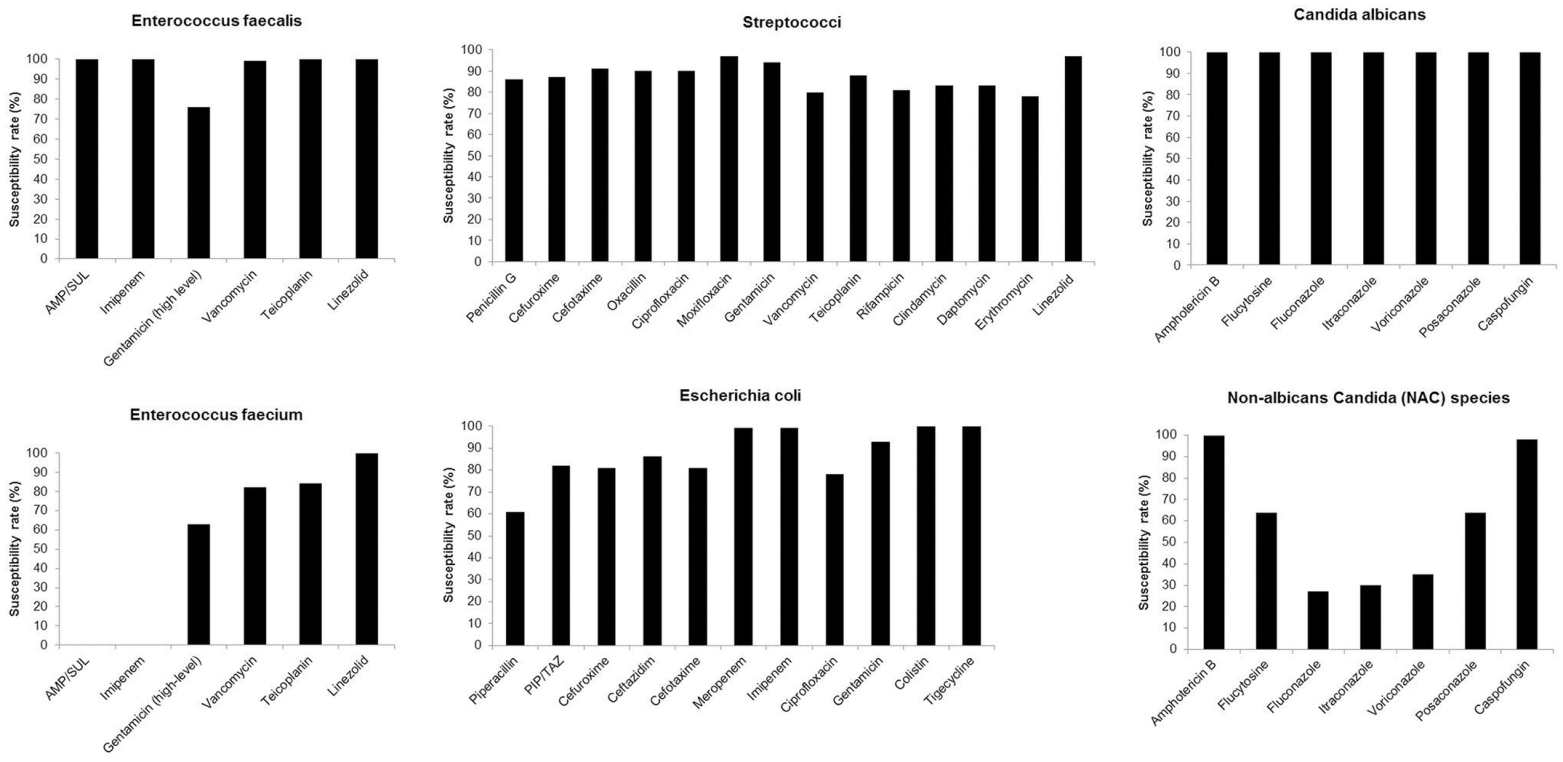
In vitro antibiotic susceptibilities of bacterial and fungal isolates, grouped by *Enterococcus faecalis*, *Enterococcus faecium*, streptococci, *Escherichia coli*, *Candida albicans*, and non-albicans *Candida* (NAC) species. Minimum inhibitory concentrations (MICs) were determined employing Industrial Organization for Standardization (ISO) 20766–1 or E-test, and the results were interpreted according to current European Committee on Antimicrobial Susceptibility Testing (EUCAST) breakpoints (www.eucast.org, 2015 edition).

Antimicrobial resistance profiles, grouped by *Enterococcus faecalis*, *Enterococcus faecium*, streptococci, *Escherichia coli*, anaerobes, *Candida albicans*, and non-albicans *Candida* (NAC) species, are distributed in Fig 7. The rate of vancomycin resistance among enterococci was 13.7% (30 of 219 isolates; 95% CI: 9.4% to 19%; 0.9% in *Enterococcus faecalis* and 28.6% in *Enterococcus faecium*). The proportion of ESBL producers among *Enterobacteriaceae* with co-resistance to ciprofloxacin was 13.9% (32/230; 95% CI: 9.7% to 19.1%). There was only one patient with the detection of carbapenem-resistant isolates due to known intestinal colonization by a *Klebsiella pneumoniae* carbapenemase (KPC)-producing *Klebsiella pneumoniae* strain, which was also detected on a biliary stent. Azole-resistant *Candida* species accounted for 32.9% (48/146; 95% CI: 25.3% to 41.1%) of the isolates.

### Independent predictors for colonization with enterococci and *Candida spp*.

Logistic regression analysis (using the following independent variables: age, gender, malignant biliary strictures, preceding liver transplantation, presence of diabetes mellitus, repeated ERC, prolonged antibiotic therapy >48 hours, and length of hospital stay >7 days) revealed that repeated ERC intervention was an independent predictor for the occurrence of enterococci (odds ratio [OR] 9.75; 95% CI: 1.24 to 76.43), and presence of a malignant biliary stricture was an independent predictor for the occurrence of *Candida spp*. (OR 2.55; 95% CI: 1.08 to 6.05).

## Discussion

In this study, 213 stents from 120 patients with the detection of 95 different bacteria and 13 different fungal species were included in the final analysis. Compared to previous analyses with a similar design and methodology [17–20,24], our study is distinguished by the large sample size; the use of sonication facilitating a better release of pathogens from the biofilm on the inner surface of the stents [10,29,30]; the application of MALDI-TOF mass spectrometry for precise identification of microorganisms; and a detailed antimicrobial susceptibility analysis involving 28 antibiotics and seven antifungal agents.

Our results reveal the highest detection rate for both enterococci (79.3% of all stents) and *Candida* species (55.9% of all stents) of all studies investigating patients with biliary stents [10,17,19,20,23–27,35–37]. The occurrence of both pathogen groups was significantly associated with an increased indwelling time of stents (Fig 5). Moreover, previous antibiotic therapy seems to exert significant selective pressure as also reported elsewhere [25,27]. Thus, the stent colonization differed significantly with respect to the proportion of *Candida* species (46.7% vs. 63%, *P* = 0.023) in patients receiving earlier prolonged antibiotic therapy. Unquestionably, this pathogen shift has clinical relevance, as conventional doctrines regarding the microbiology of the biliary tract describe a predominance of *Enterobacteriacea*e and anaerobes, followed by enterococci and streptococci [38,39].

Preceding studies have shown a clear association between biliary stent insertion and changes in the spectrum of pathogens in the bile toward enterococci [10,23,36], as well as the increased incidence of bacterial and fungal colonization associated with pre-operative biliary drainage [37]. In a study performed by Weber et al. in Germany, enterococci were by far the most prevalent genera, reaching detection rates of >70% in patients with stent-associated cholangitis [23]. Thus, the high proportion of enterococcal species of 79.3% in our study is in line with previous reports, revealing that samples collected from patients with stents had a significantly higher incidence of *Enterococcus spp*. compared to bile samples from patients without stents, corroborating a “stent tropism” of enterococci [10,23,36]. Furthermore, the number of enterococci isolated from blood cultures appeared to be higher in stent-associated cholangitis episodes [23]. In this context, repeated ERC intervention was an independent predictor for the occurrence of enterococcal species (OR 9.75) in our study. The well-described risk factors of *Candida* in bile include biliary stenting, malignant strictures, and repeated interventions [23,26]. In such patients, prolonged antibiotic treatment apparently facilitates biliary colonization by *Candida spp*. [26,27], as also shown in our study. From a clinical point of view, the detection of significantly more *Candida spp*. (as well as viridans group streptococci) in patients with malignant disease may also reflect a certain degree of tumor-associated immunological dysfunction.

The majority of stent extractions were performed electively, and therefore, blood cultures were collected only in patients presenting with acute cholangitis or general signs of infection. Because the matching rate of blood cultures and stent isolates was 56.7% in our study, we assume that corresponding shifts in the biliary pathogen spectrum toward enterococci and *Candida spp*. may be clinically relevant in the case of stent-associated acute cholangitis. However, Schneider et al. already pointed out that the hypothesis that microorganisms isolated from the biofilms on biliary stent surfaces are similar to microorganisms in the bile fluid still has to be proven [10], as concomitant bile cultures were not systematically collected in most available studies and systematic microbiological typing was omitted. Negm et al. concluded in a recent study of endoscopically obtained bile aspirates that all microorganisms found in positive blood cultures were also found in bile samples [26], indicating that biliary culture results alone are as effective as positive blood cultures. In the literature, the clinical relevance of endoscopically performed bile collection for microbiological analysis is discussed controversially [10,26,35–37]. In a study performed by Park et al. in Korea, in 258 bacteremic cholangitis episodes, complete agreement with blood cultures was observed only in 31% of the bile samples [35]. Fifty percent of the bile specimens showed partial agreement with the blood culture findings, and 19% of the bile cultures revealed completely different microorganisms compared to the blood cultures analyzed. The degree of coincidence between the bile and blood cultures for *Escherichia coli*, *Klebsiella spp*., enterococci, and streptococci was 71%, 53%, 35%, and 27%, respectively [35]. In our setting, the risk of cholangitis was significantly higher in patients with stent occlusion (38.5% vs. 9.1%, *P* <0.001). In the study performed by Schneider et al., biliary stents occluded after a median indwelling time of 70 days [10]. However, stent occlusion resulted in cholangitis or cholestasis in only 35% of the cases. Thus, stent occlusion is not always associated with clinical symptoms.

The question of the optimal stent surface with regard to the prevention of occlusion has not yet been answered satisfactorily, whereby an advantage for Teflon^™^ (polytetrafluorethylene) compared to other plastics could be demonstrated [6]. Several studies have shown that antimicrobial coatings on medical devices are effective against the formation of microbial biofilms [10]. Therefore, the use of special coatings, such as diamond-like carbons (DLC) or silver-nanoparticles, seems to be a promising approach for reducing biofilm formation on medical implants [40,41]. On the other hand, there has been no evidence so far that modified surfaces offer effective protection against the apparently growing threat of colonization by enterococci and *Candida* species.

Regarding antimicrobial resistance profiles, in our environment, resistance rates to vancomycin in enterococci (0.9% in *E*. *faecalis*, and 28.6% in *E*. *faecium*), the rate of co-resistance to ciprofloxacin in ESBL-producing *Enterobacteriaceae* (13.9%), and the resistance rate to carbapenems in *Enterobacteriaceae* (0.4%) were still within feasible ranges. However, taking into account the azole resistance rate of 32.9%, significant failure rates of fluconazole-based empirical antifungal therapies for *Candida* infections have to be considered. It is well known that numerous interventional ERC procedures accompanied by antibiotic therapy predispose for increased antimicrobial resistance in patients with acute cholangitis [25,27], and enterococcal species are often involved in this process [42,43]. Therefore, adequate microbiological diagnosis is essential and should include analysis of the extracted stents.

Multi-drug resistance is generally on the rise, particularly among *Enterobacteriaceae* [44–47]. Regarding the detection of carbapenem-resistant *Enterobacteriaceae* (CRE) in a single patient in our study, it is important to keep in mind that biliary colonization with CRE may have enormous clinical relevance in certain patient groups, such as liver transplant recipients [48]. Taking into consideration existing hygiene recommendations for the clinical management of affected patients in Germany, we propose active surveillance and stringent contact isolation precautions in hospitals, particularly in the endoscopy unit [49]. Invasive procedures as well as the use of antibiotics should be limited to clear medical indications.

In the overall assessment, our study has limited representativeness due to its monocentric design, and it displays several limitations: Firstly, the contamination of samples during endoscopic retrieval of stents cannot be excluded. Secondly, the choice and indwelling times of polyethylene or polyurethane stents inserted in the bile duct were arbitrary. Therefore, the timing of stent extraction and the indications for stent placement were not standardized. Thirdly, clinical selection bias cannot be excluded because it was a non-randomized study, and the results of the microbiological analysis are certainly influenced by the use of antimicrobial agents.

## Conclusions

We conclude that enterococci and *Candida* species play an important role in the microbial colonization of biliary stents. Therefore, empirical antimicrobial treatment of stent-associated cholangitis should be guided toward enterococci, *Enterobacteriaceae*, streptococci, anaerobes, and *Candida*. To determine causative pathogens, an accurate microbiological analysis of the extracted stent(s) may be helpful. Assessing the presence of pathobionts versus symbionts in the biliary tract is an important task of future clinical studies.

## Supporting Information

S1 Dataset The study data base is available for download. (XLSX)

S1 Table Microorganisms isolated from biliary stents in relation to disease and stent characteristics. (DOC)
