# Supplementary material for: Epidemiology and Resistance Patterns of Bacterial and Fungal Colonization of Biliary Plastic Stents: A Prospective Cohort Study
Source: PLoS One. 2016 May 12;11(5):e0155479. doi: 10.1371/journal.pone.0155479 (PMC4865241; doi:10.1371/journal.pone.0155479)
Supplement: S1 Table — (DOC) [file pone.0155479.s002.doc]

***S1 Table:*** Microorganisms isolated from biliary stents in relation to disease and stent characteristics

| **Microbial species** | **Malignant vs. benign (%)** | | **Occluded vs. non-occluded (%)** | | **Straight vs. double pigtail (%)** | | **Cholangitis vs. colonization only** | |
| --- | --- | --- | --- | --- | --- | --- | --- | --- |
| Enterococci | 59/76 (77.6%) | 110/137 (80.3%) | 20/26 (76.9%) | 149/187 (79.7%) | 75/100 (75%) | 94/113 (83.2%) | 21/27 (77.8%) | 148/186 (79.6%) |
|  | *P* = 0.724 | | *P* = 0.796 | | *P* = 0.175 | | *P* = 1.00 | |
| *Candida* | 51/76 (67.1%) | 68/137 (49.6%) | 18/26 (69.2%) | 101/187 (54%) | 54/100 (54%) | 65/113 (57.5%) | 13/27 (48.1%) | 106/186 (57%) |
|  | *P* = 0.015 | | *P* = 0.205 | | *P* = 0.679 | | *P* = 0.413 | |
| *Entero-bacteriaceae* | 56/76 (73.7%) | 101/137 (73.7%) | 18/26 (69.2%) | 139/187 (74.3%) | 71/100 (71.0%) | 86/113 (76.1%) | 21/27 (77.8%) | 136/186 (73.1%) |
|  | *P* = 1.00 | | *P* = 0.636 | | *P* = 0.437 | | *P* = 0.651 | |
| Streptococci | 31/76 (40.8%) | 36/137 (26.3%) | 10/26 (38.5%) | 57/187 (30.5%) | 31/100 (31.0%) | 36/113 (31.9%) | 8/27 (29.6%) | 59/186 (31.7%) |
|  | *P* = 0.032 | | *P* = 0.499 | | *P* = 1.00 | | *P* = 1.00 | |
| Staphylococci | 10/76 (13.2%) | 14/137 (10.2%) | 3/26 (11.5%) | 21/187 (11.2%) | 15/100 (15.0%) | 9/113 (8.0%) | 3/27 (11.1%) | 21/186 (11.3%) |
|  | *P* = 0.652 | | *P* = 1.00 | | *P* = 0.130 | | *P* = 1.00 | |
| *Pseudomonas* | 1/76 (1.3%) | 12/137 (8.8%) | 3/26 (11.5%) | 10/187 (5.3%) | 7/100 (7%) | 6/113 (5.3%) | 3/27 (11.1%) | 10/186 (5.4%) |
|  | *P* = 0.035 | | *P* = 0.376 | | *P* = 0.776 | | *P* = 0.380 | |
| Anaerobes | 18/76 (23.7%) | 30/137 (21.9%) | 7/26 (26.9%) | 41/187 (21.9%) | 21/100 (21%) | 27/113 (23.9%) | 5/27 (18.5%) | 43/186 (23.1%) |
|  | *P* = 0.864 | | *P* = 0.617 | | *P* = 0.627 | | *P* = 0.637 | |
